# Perspectives on health care transition among U.S. parents of internationally adopted children living with HIV: A qualitative study

**DOI:** 10.1016/j.hctj.2023.100010

**Published:** 2023-07-14

**Authors:** Faith Glover, Rosemary Olivero, Cynthia Fair

**Affiliations:** a Elon University, Elon, NC, USA; b Department of Pediatric Infectious Disease at Helen Devos Children’s Hospital of Spectrum Health, Grand Rapids, MI, USA; c Public Health Studies at Elon University, Elon, NC, USA

**Keywords:** International adoption, Pediatric HIV, Adoptive parents, Health care transition, Qualitative methods

## Abstract

**Objective:**

To analyze perspectives on health care transitions among parents who have internationally adopted children living with HIV (IACH)The transition of youth with living with HIV from pediatric to adult care is associated with adverse health outcomes, including poor medication adherence and appointment attendance. However, little is known about the experiences of IACH.

**Methods:**

This qualitative project explores the perspectives of 17 parents of 24 IACH in the United States through hour-long semi-structured phone interviews focused on healthcare transition. The purposive sample was recruited from two pediatric infectious disease clinics and private social media sites. Drawing on analytic principles of constant comparison, transcripts were analyzed for emergent themes.

**Results:**

Most parents identified as white (*n* = 16), female (*n* = 16). Median age of IACH was 16 years. Two had transitioned to adult care. Fourteen did not have a transition plan with their provider. Many parents expressed apprehension regarding the transition to adult care. Anxiety over the ability to communicate with their child’s health provider and lack of comprehensive planning were expressed. Parents also felt their child may feel out of place in the adult infectious disease clinic and emphasized the trusting, long standing relationship with pediatric providers. Participants acknowledged that transition to another provider could be challenging for their child as adult providers may be less aware of adoption-related trauma.

**Conclusion:**

It is vital that physicians consider trauma-informed care throughout the transition process with IACH. Providers should support health management-related independence of both IACH and their parents prior to transition. Coordination with adult care providers is key to a successful health care transition.

## 1 Introduction

In 2010, the ban of human immunodeficiency virus (HIV)-positive individuals immigrating into the United States was repealed.^1^ This act has since allowed for the adoption of children with HIV, and the population of internationally-adopted children living with HIV (IACH) has grown. Limited information exists on IACH, with only one study focusing on health outcomes of the population in the United States. Wolf et al.^2^ conducted a retrospective chart review of 79 HIV-positive international adoptees or refugees in foster care (mean age: 6.1 years, range 3.7–8.6 years) at two hospitals in Colorado and Washington state. Countries in Africa (90%) were the primary region origin for study population. At the time of adoption, 75% of participants were on antiretroviral therapy (ART), with the majority of those (76%) virally suppressed. Half of the adoptees had educational delays and 48% had one or more mental health or behavioral problems.

Individuals living with HIV have traumatic experiences at disproportionately higher rates than individuals who are not living with HIV.^3^ In the case of internationally-adopted youth with HIV in particular, Wolf et al.^2^ found that 24% of participants had a diagnosed mental health condition. Peck et al.^4^ found that adverse childhood experiences are linked with lower ART adherence and higher levels of substance use in individuals living with HIV. Further, attachment disorders and behavioral issues are common in adopted children with trauma histories, with 74% of parents reporting that their adopted child had challenging behaviors.^5^ This population in particular may be faced with a unique set of experiences.^6^ In addition to living with HIV, all experienced abandonment and many lost one or both biological parents prior to being adopted.

### 1.1 Health care transition

Health care transition is the process when an adolescent or young adult with or without special healthcare needs moves from a pediatric to an adult health care provider.^7^, ^8^ Throughout transition, there is a change in the way health care is delivered from the more familiar environment of pediatric clinic to the unknown adult care clinic.^9^ Parents perceived pediatric care as more organized, and pediatric providers were viewed as more available for patient concerns relative to adult care.^9^

Multiple adverse health outcomes are associated with the transition of youth with HIV from pediatric to adult care including lower levels of medication adherence, lower appointment attendance, and poorer health outcomes.^10^ Differences in care between pediatric and adult care, changes in health network or financial coverage, and psychosocial issues experienced by the patient throughout the process were other factors that can inhibit a successful transition.^11^ Fair et al. found that pediatric and adult infectious disease medical providers identified best practices of transition to include supporting the medical independence of patients, early transition preparation, engaging caregivers in the process, and increasing communication between pediatric and adult providers.^12^

### 1.2 Current project

Previous research has identified that the transition to adult care is challenging for youth with special health care needs, including those with HIV.^9^, ^10^ However, no research has specifically looked at the transition of IACH who may have unique needs due to their prior traumatic experiences. The current study evaluates the anticipated or experienced challenges with transition to adult care from the perspective of parents of IACH.

## 2 Methods

### 2.1 Participants

This study focused on 17 parents of IACH living in the United States. The participants were recruited from a previous study (Helen DeVos Children’s Hospital, Seattle Children’s Hospital) as well as a private Facebook group separate from either children’s hospital.^11^ We originally gained access to this Facebook group when one of the adoptive parents recruited from a hospital clinic asked if she could post a flyer about our research project within the group. Purposive snowball sampling, as employed in Fair et al.^13^, was used to locate individuals. A digital flyer was distributed to previous participants and to the parent’s Facebook group to share the objectives of the study and invite participation. The research was approved by Elon University’s Institutional Review Board. Participants completed informed consent documentation and received a $25 gift card as a token of appreciation for their time.

### 2.2 Study design

This cross-sectional qualitative study consisted of 17 interviews with parents of IACH whose child was 12 years or older. The semi-structured interviews each lasted approximately 60 min and were conducted by two interviewers between March and August of 2021. All interviews were conducted by phone, recorded, and transcribed. Participants first were asked demographic questions. Subsequent questions centered around the transition of youth from pediatric to adult infectious disease care, as well as other issues that their child will face in adolescence such as sexuality and intimate relationships. Questions were adapted from previous research on the transition of youth with HIV to adult care.^12^ Questions were also vetted by our Community Advisory Board comprised of adoptive parents. Finally, questions were piloted to ensure clarity of the questions prior to the phone interviews. Sample questions included:

• Have you or your child’s provider discussed transition with your child? If so, please elaborate on topics covered.

• What, if any, benefits do you expect of transition?
• What, if any, concerns do you have related to transition?
• What can pediatric/adult providers do to best assist youth in preparing for transition?

### 2.3 Data analysis

After the interviews were completed, they were transcribed using a transcription service (Rev.com), uploaded into Dedoose version 9.0.46, and coded using a content analysis technique.^14^ The “Sort and Sift, Think and Shift” method was used for qualitative analysis.^15^ This methodology involves numerous rounds of “diving in” to analysis and then “stepping back” to gain a more holistic perspective of the research at the moment. Additionally, episode profiles were made for each transcript and researchers summarized the themes as they emerged. Initial interviews were coded to inform subsequent interviews. Authors FG and CF read through all transcripts and participated in primary content analyses. Research team members met weekly during data collection to discuss themes and to come to consensus on themes across transcripts.

## 3 Results

This study included 17 parents of IACH. Adoptive parents were from ten states across the U.S. Sixteen identified as white and female with a mean age of 45.3 years. Additional demographic information about participants can be found in Table 1.

**Table 1:** Parent Demographics (*N* = 17).

Gender Female Male	16 1
Race White Black	16 1
Age (years)	45.3 (range: 43–55)
Marital Status Married	17
Biological Children Yes No	16 (mean number of biological children: 2.50, range 1–5) 1
Adopted Children Yes	17 (mean number of adopted children: 2.75, range: 1–7)
Number of IACH/family 1 2 3	11 5 1

The 24 IACH were adopted from various countries, including Ethiopia (*n* = 9), Uganda (*n* = 2), Ukraine (*n* = 3), India (*n* = 2), Russia (*n* = 2), Vietnam (*n* = 2), Georgia, Ghana, Haiti, and Sierra Leone. Thirteen identified as female and the median age was 16 years. See Table 2 for demographic features of adoptees.

**Table 2:** IACH Demographics (*N* = 24).

Gender Female Male	13 11
Age at time of Adoption	Median 7 years (range: 4–15)
Age at time of Interview	Median 16 (range: 12–24)

### 3.1 Qualitative findings

Analyses revealed three primary themes related to health care transition: trauma, lack of transition preparation, and differences in pediatric and adult care.

### 3.2 Trauma

The complex nature of IACH identities meant that all the children in this cohort had traumatic experiences in their country of origin prior to adoption. All had lived in an orphanage or temporary care due to the death of one or both parents. Some had family members living in their country of origin who refused to care for a child living with HIV. Further, children were internationally adopted after these significant childhood experiences. Such experiences contributed to attachment disorders and behavioral issues, which were common in our sample. Subthemes include trauma in the child’s country of origin, adoption-related trauma, and attachment issues.

“HIV basically ravaged the family.” As one mother of an 18-year-old daughter stated, it is “just the nature of our children, they are coming to us with trauma and loss and providers need to know that.” IACH had numerous family members pass away due to AIDS-related causes, and had early lives marked with death. One participant described,

… her mother and father had HIV and when the mother found out she killed herself because of the stigma. And then the father died of AIDS shortly after. (mother of 16-year-old female.

Another shared symptoms due to trauma experienced in the child’s country of origin, “He was having night terrors and just lots of memories back to when his mom died, and when he thought he could help her and save her. I think he was nine. He watched her die.”

“Adoption-related trauma is real.” In addition to trauma in the child’s country of origin, parents noted that the simple act of adoption brings with it challenges. For example, one parent discussed the identity issues faced by his son, and how they are distinct from the challenges of their other adopted children,

…he's had many kinds of identity issues… most of the kids that we have, have a sibling or they know who their parents were, and that's much more prevalent in his life, a feeling that he doesn't have a sibling, and he's not been able to locate his mother. (mother of 17 year-old male).

Parents noted the importance of finding mental health clinicians with expertise in adoption-related trauma was a significant challenge. One parent explained:

We know that they are probably going to need to access mental health services for most of, if not all of their lives, and when you throw on top of that HIV stigma, the research is there that those living with HIV have higher incidence of depression and anxiety because of it as well. So, we need to make sure our kids are set up for success. (mother of 16 and 18-year-old females).

“That’s been our biggest heartache… attachment is a huge issue. HIV has just been a disease.”

Multiple aspects of the complex identities of IACH, being both internationally adopted and living with HIV, can contribute to attachment disorders for youth in this population. One parent explained how attachment issues were impacting both the parent-child relationship as well as his son’s medical coverage,

My son says, “I don't need a family. I don't want a family. I don't want parents. You're not my parents. You didn't ask me if I wanted to be adopted”…he wanted to be taken off our insurance… You would think me keeping him on my insurance is a normal and nice thing. He says I'm being selfish by keeping him on my insurance. That's his attachment disorder speaking. (father of 18-year-old male)

Attachment disorders are also relevant for any medical provider(s) interacting with this population, as it can alter how IACH connect with others and receive care. Youth may be mistrustful of a new health care provider. One parent discussed how his child’s issues were impacting his ability to smoothly transition from pediatric to adult care,

So how does that play into his transition? He's extremely at-risk attachment disorder kids and you know, it starts as a bipolar thing. There's like a slide that starts as ADHD or bipolar, but it actually has delusions of grandeur, delusions of independence. Do you know anybody in their right mind that would want to get taken off insurance? Like, dude, I'm paying for you for free. (father of 18-year-old male).

Parents also commented on the concern that adult providers would not know how to treat youth with significant trauma histories and attachment issues. One parent stated, “I’m just really nervous. I mean are they going to be able to effectively treat my child who has reactive attachment disorder? It seems unlikely.” Another parent said, “Children with attachment disorders have unique needs and I’m not certain the adult clinic providers are trained in how to engage someone with that diagnosis.” Parents specifically noted the need for trauma-informed care. A parent noted, “Adult providers need to know about trauma-informed care. Our pediatrics team certainly practices trauma-informed care. I worry the adult care clinicians will be too busy or not trained at all in this practice.”

### 3.3 Lack of transition preparation

Parents described that both they and their IACH were underprepared for transition and emphasized the important developmental considerations that could play a role in the transition process. Subthemes include lack of communication and child readiness.

“I don’t know if she just stops at age 18. I have no idea. We’ve not talked about it.” Many parents shared that transition had not yet been discussed with the pediatric provider. In fact, over half of IACH age 16 or older had not yet talked about transition with their provider. One participant described,

I haven’t made any changes just because not making changes is easier than making changes…I’ve been leaving it up to the doctor’s office. They know he’s 19 and they haven’t said one word about the transition yet…(mother of 19-year-old male)

Another parent of an 18-year-old male noted, “We haven’t heard a word about transition.” Parents also shared that they had not yet thought much about the logistics of transition and still had questions regarding timeline and adult care providers. However, transition was often not an area of major concern for parents of younger adolescents, as many felt as if they had time to learn more about the process. For example, one participant stated,

…in Michigan, we have this children’s special health care, which is a program that covers all of their medications, all their HIV meds and anything, any medical costs related to HIV. And I don’t even know how long that goes. Does it end at age 18 or age 20? So those are things that I probably should eventually look into, but like I said, she’s 14. So I just feel like I do have time. (mother of 14-year-old female)

“There are some things that she’s just not ready to do on her own.” Parents shared concerns about their IACH transitioning, as they felt that they may not be ready to manage their care independently. Many indicated that their child should slowly increase personal health responsibility through activities such as picking up medication, going to the clinic alone, etc. One participant described their adolescent,

For example… the pharmacy… she wouldn’t be responsible at this point to get her prescriptions from the pharmacy to do those refills. So that’s something that mom still probably needs to do for a couple of years. (mother of 19-year-old female).

Several parents expressed apprehension of the age for transition. One mother stated, *“*Their development is not all the same just because they magically turn 18 or 21 or whatnot.”

The age cutoff for transition differed based on hospital/clinic or coverage regulations, most frequently being 18, 22, or 25. The age of transition seemed to be arbitrary and without reasoning as to age differences across clinics. Although parents perceived that their child would not be ready to transition at the expected age, it may be a forced due to policies around required age of transition. One mother of a 19-year-old daughter relayed the following story,

She’s been going to her medical appointments with her pediatric ID team on her own since she turned 18. One day she returned from the appointment to say that she had transitioned to the adult clinic. No warning, no process, no nothing.

Another parent explained, “She’s already transitioned to the adult clinic, but I have to remind her make and keep the appointments. I think she’ll always need some support.”

Multiple parents argued for a gradual transition based upon developmental readiness, rather than age. For example, one parent felt her 16-year-old child would not be ready to transition at 18 because, “my daughter has bipolar. Her poor organization skills and her mania and depression [affect] her maturity levels. She can get her bloodwork done but needs help with her medication. This means parents should be highly involved in that transition.”

### 3.4 Perceived differences between pediatric and adult care

Another major topic of discussion was the perceived stark contrast between provider care and the environment in pediatric versus adult health care. Pediatric clinics were associated with support and access to psychosocial services. Several parents expressed concern that their child would not “fit in” in the adult care clinic. Subthemes include comfort in the pediatric clinic and fear of adult care.

“We’re really happy at the clinic.” The majority of parents emphasized the positive, trusting relationship their IACH had with their pediatric provider. Most parents said that their child would stay in pediatric care as long as allowed, one stated “I can’t imagine her transitioning any sooner than she has to.” For example, one parent described the close connection established with the pediatric infectious disease provider formed over time, saying,

…trust is probably the best word. You sort of put in that relationship that you've had for so many years. I mean, she already advised us before he even came home, on his care…we were able to make some changes to his medical care while he was still in Ethiopia based on her medical advice. So, she's been with us for a long time. (mother of 14 year-old male)

Many IACH in the study had been with the same pediatric provider for the entirety of their care in the United States, so the patient-provider bond was strengthened by the longstanding nature of care received. One parent described how her child was deeply attached to the staff at the pediatric clinic,

… she's got people who've seen her graduate kindergarten, graduate middle school, graduate high school, you know what I mean?. They've been through every stage of her life. They know her. (mother of 18-year-old female)

Further, pediatric providers often played a role in disclosure to the child. All children over the age of 12 knew their HIV status.

“I don't want to transition to that clinic.” Most parents described that both they and their IACH wanted to postpone transition as long as possible due to uncertainty and fear surrounding adult infectious disease care. Some parents described concerns about different patient expectations in adult care, as not only would providers not be used to treating an IACH patient but also their child would be surrounded by a different demographic in the clinic. For example, one mother recalled a call with an adult clinic,

…there's one place where [doctor] sees pediatric clients where also adults are seen. And when I called to schedule, somebody said to me, ‘well, you might not want to, it's maybe not the place you want to be with your young child.’…I just know they're gonna be lumped in with a whole different demographic once [transition] happens… But, just talking about identity and the perception of your own illness, I would say, when all those things are affected by the care you receive and how you receive it certainly. (mother of 12-year-old male)

Another parent was concerned that her son would need to attend, “the clinic would be for people who, you know, acquired HIV, you know, as teens or young adults and you know, where my son acquired it at birth. I don't want to transition to that clinic.”

Additionally, there was a common perception of adult care as being less supportive and structured for patients. The community and more abundant psychosocial resources found in pediatric care were most often listed as reasons for wanting to delay IACH transition to adult care. One parent stated,

You know, in adult care, it's different there. You don't have a doctor, a social worker, a nurse, a case manager… you don't have a medical care model. They don't text and call you and send you birthday cards…I mean, you're just another patient. They have an automated thing call you once a year. If you show up, you show up. If you don't, you don't. Whatever. (mother of 24-year-old female).

## 4 Discussion

Transition from pediatric to adult health care is an important and necessary process in the lives of IACH. The purpose of this study was to explore the experiences and opinions of parents of IACH, and to gain an understanding of parental perspectives in order to inform future practices in the transition of IACH from pediatric to adult care.

The current study found that parents believed attachment disorders and trauma may adversely influence transition to adult care. This is a unique finding in the literature on transition to adult care within the population of youth with HIV. Due to the significant impacts that trauma and attachment play in the lives of IACH, it is vital that providers account for this in their care. Research suggests that adult who have a history of attachment disorder may have difficulty connecting with their adult providers.^16^ Further, Lan Lee et al. found that adults with a history of adverse childhood events have increased reporting of symptoms and health anxiety.^17^ Many parents had concerns that adult care providers would not know how to treat youth with the unique trauma and attachment styles commonly found in IACH.

Tanner at al. described the potential usefulness of pediatric-specific training for adult providers caring for youth after transition.^18^ Specific to the population of IACH and based on the results of this study, it would be useful to train adult providers about potential attachment issues in IACH as well as in trauma-informed care. Training may improve long term retention in adult care for IACH as well as prove pertinent for pediatric providers, as they initiate and facilitate the transition of this population. Peck et al.^4^ called for regular screening of adverse childhood experiences for adults living with HIV in a primary care setting. Such screening may be useful for youth transitioning into adult care. This is particularly relevant to IACH who may have attachment issues that interfere with transition such as establishing a trusting relationship with a new provider and increased self-management requirements.

Trauma-informed care was described by parents in the current study as a useful approach and may help reduce negative health consequences frequently found in individuals with childhood trauma. Researchers have argued that trauma-informed care is valuable for adults living with HIV.^19^ However, Piper et al. found that significant barriers exist to consistently providing trauma-informed care across HIV treatment centers.^20^ Challenges include lack of staff training and resources to address identified needs.

Most parents shared that they did not feel as though they or their child were well prepared for the transition to adult care. Results indicate a lack of discussion or planning of both providers and parents. Similarly, Fair et al.^21^ found that few U.S. parents of adolescents with HIV had discussed transition with their child. The majority of parents, even those of teenagers, shared that their child’s provider had not yet discussed transition with them at all, and they had no plans in place. Previous research confirms a noted lack of provider communication around transition among adolescents with HIV.^12^ Parents also described feeling as though they had plenty of time to prepare for transition, and that it would be an issue they would tackle closer to the time it would occur.

The current study also reinforced the difference in parental perceptions that pediatric care is viewed as a more supportive and positive environment relative to adult care. All parents explained that their IACH planned on staying with their pediatric provider as long as allowed, often citing the well-established patient-provider relationship as a primary reason for doing so. This follows the results of Lam et al.’s research in the UK, which found that the major barriers to a successful transition for adolescents living HIV all revolved around the significant change in support and culture from pediatric to adult care.^22^ The fear surrounding different patient ages and routes of transmission at adult clinics and that adult providers may not be accustomed to providing the necessary support to newly transitioned youth with HIV was also confirmed by Kakkar et al.^23^ Results also indicated that some parents may hold be stigma against other people living with HIV. This should be addressed during the transition process as such biases could negatively affect a successful transfer to adult care.

One primary way that the transition process of IACH can be improved is early preparation and planning.^24^ Similarly, Heath et al.^.^ described the benefit of parents encouraging their child’s health autonomy prior to transition. ^25^ Parents in our study described providing their child with specific healthcare tasks, such as picking up medication or attending appointments alone, in order to slowly build their responsibility over time. It would be beneficial for providers to support health management-related independence of both IACH and their parents prior to transition due to the high level of involvement of parents in the healthcare of their IACH. Coordination and communication between parents and providers is key to a successful health care transition with the goal of continued medication adherence and engagement in adult care.^12^

## 5. Limitations

While the results of this study contribute clinically meaningful information about health care transition of IACH, there were several limitations that should be noted. Primarily, the sample of this study was relatively uniform in nature. All IACH were on ART and were virally undetectable and well connected to care which limits generalizability. Further, selection bias could exist in our sample. For example, families who have IACH are likely higher resourced than refugees or part of undocumented families. Only two of the IACH had already transitioned to adult care and the median age of participants was 16 years. Therefore, the majority of the IACH in this group were at the beginning of their transition journey. Findings mostly represent the anticipatory views of adoptive parents. Additionally, the sample of parents interviewed for this study was relatively homogenous and could be a result of selection bias. Further, the sample size is relatively small. While it was beneficial to hear parental perspectives on transition, the outlook of IACH was not included. Future research should aim to speak directly to adopted youth before, during, and after transition, as well as hear provider perspectives on how to best encourage effective transition preparation and care for this population.

## 6 Strengths

Despite limitations, this is, to our knowledge, the first study of perceptions of health care transition to among parents of IACH. Findings can inform interventions designed to promote a successful transition to adult care. While adoptees are likely to experience the same kind of apprehensions that non-adopted youth have, concerns are compounded by a history of trauma and disrupted parent-child attachment. The qualitative methodology allowed the capture of the lived experiences of adoptive parents.

## 7 Conclusion

Our study sheds light on perceptions of the inevitable move from pediatric to adult-focused care among a unique and vulnerable population. Providers should support the health management-related independence of both IACH and their parents prior to transition while being mindful of the role that previous trauma may play in their comfort with transition to adult care.^12^, ^17^ Coordination with adult providers is key to a successful health care transition.

## Funding

This research was funded by the 10.13039/100006826Elon University Honors Fellows and the Summer Undergraduate Research Experience programs.

## CRediT authorship contribution statement

All authors on this paper meet the four criteria for authorship as identified by the International Committee of Medical Journal Editors (ICMJE); all authors have contributed to the conception and design of the study, drafted or have been involved in revising this manuscript, reviewed the final version of this manuscript before submission, and agree to be accountable for all aspects of the work. Specifically, using the CRediT taxonomy, the specific contributions of each author is as follows: Conceptualization & Methodology: Faith Glover, Rosemary Olivero, Cynthia Fair, Formal analysis: Faith Glover, Cynthia Fair, Funding acquisition: Faith Glover, Cynthia Fair, Investigation: Faith Glover, Cynthia Fair, Project administration: Rosemary Olivero, Cynthia Fair, Supervision: Cynthia Fair, Validation: Faith Glover, Rosemary Olivero, Cynthia Fair, Original draft: Faith Glover, Cynthia Fair, Writing/Revising – Faith Glover, Rosemary Olivero, Cynthia Fair.

## Declaration of Competing Interest

The authors declare that they have no known competing financial interests or personal relationships that could have appeared to influence the work reported in this paper.

## Data Availability

Data will be made available on request.
